# Evolutionary origins of abnormally large shoot sodium accumulation in nonsaline environments within the Caryophyllales

**DOI:** 10.1111/nph.14370

**Published:** 2016-12-05

**Authors:** Philip J. White, Helen C. Bowen, Martin R. Broadley, Hamed A. El‐Serehy, Konrad Neugebauer, Anna Taylor, Jacqueline A. Thompson, Gladys Wright

**Affiliations:** ^1^ The James Hutton InstituteInvergowrieDundeeDD2 5DAUK; ^2^ Distinguished Scientist Fellowship ProgramKing Saud UniversityRiyadh11451Saudi Arabia; ^3^ Warwick HRIUniversity of WarwickWellesbourneWarwickCV35 9EFUK; ^4^ Plant and Crop Sciences DivisionUniversity of NottinghamSutton BoningtonLoughboroughLE12 5RDUK; ^5^ Zoology DepartmentCollege of ScienceKing Saud UniversityRiyadh11451Saudi Arabia

**Keywords:** Aizoaceae, Amaranthaceae, Caryophyllales, halophyte, hyperaccumulation, phylogeny, shoot, sodium (Na)

## Abstract

- The prevalence of sodium (Na)‐‘hyperaccumulator’ species, which exhibit abnormally large shoot sodium concentrations ([Na]_shoot_) when grown in nonsaline environments, was investigated among angiosperms in general and within the Caryophyllales order in particular.
- Shoot Na concentrations were determined in 334 angiosperm species, representing 35 orders, grown hydroponically in a nonsaline solution.
- Many Caryophyllales species exhibited abnormally large [Na]_shoot_ when grown hydroponically in a nonsaline solution. The bimodal distribution of the log‐normal [Na]_shoot_ of species within the Caryophyllales suggested at least two distinct [Na]_shoot_ phenotypes within this order. Mapping the trait of Na‐hyperaccumulation onto the phylogenetic relationships between Caryophyllales families, and between subfamilies within the Amaranthaceae, suggested that the trait evolved several times within this order: in an ancestor of the Aizoaceae, but not the Phytolaccaceae or Nyctaginaceae, in ancestors of several lineages formerly classified as Chenopodiaceae, but not in the Amaranthaceae *sensu stricto*, and in ancestors of species within the Cactaceae, Portulacaceae, Plumbaginaceae, Tamaricaceae and Polygonaceae.
- In conclusion, a disproportionate number of Caryophyllales species behave as Na‐hyperaccumulators, and multiple evolutionary origins of this trait can be identified within this order.

## Introduction

Sodium (Na) is not considered to be an essential element for plants (White & Brown, 2010), although it is required (in micronutrient quantities) for the C_4_ photosynthetic pathway (Cheeseman, 2015) and some halophytes (euhalophytes) grow better when supplied with Na (Greenway & Munns, 1980; Albert, 1982; Flowers & Colmer, 2008; Munns & Tester, 2008; Rozema & Schat, 2013). In addition, in some environments, for example where there is low K^+^ phytoavailability, plant growth can benefit from a source of Na, as Na^+^ can replace K^+^ as a cationic osmoticum in the vacuole (White, 2013). The accumulation of excessive Na concentrations in plant tissues is, however, detrimental to plant growth as Na^+^ interferes with metabolism in the cytoplasm, mitochondria and plastids (Flowers *et al*., 2015).

It is estimated that > 6% of the world's land, and 5–15% of the world's agricultural land, is adversely affected by Na concentration through either salinity or sodicity (Munns & Tester, 2008). Saline soils are generally dominated by NaCl, although there are often significant concentrations of Ca^2+^, Mg^2+^, ${\text{SO}}_{4}^{2-}$ and ${\text{CO}}_{3}^{2-}$. They are defined as having < 15% of their exchangeable cations as Na^+^ and soil solutions with electrical conductivity (EC_e_) > 2 dS m^−1^ in a saturated paste extract, which equates to an NaCl concentration of 20 mM and pH < 8.5. Sodic (alkali) soils are generally dominated by Na_2_CO_3_, and are defined as having > 15% of their exchangeable cations as Na^+^ and soil solutions with EC_e_ > 2 dS m^−1^ and pH > 8.5 in a saturated paste extract. Saline‐sodic soils have > 15% of their exchangeable cations as Na^+^ and soil solutions with EC_e_ > 2 dS m^−1^ and pH < 8.5 in a saturated paste extract.

Halophytes are generally defined as plants that inhabit saline environments or that complete their life cycles in the presence of large concentrations of ions (≥ 200 mM), most commonly NaCl, in the root zone (Flowers & Colmer, 2008). They can be further classified into miohalophytes, which exhibit maximal growth in nonsaline environments, and euhalophytes, which exhibit maximal growth under saline conditions (Greenway & Munns, 1980). Halophytes tolerating EC_e_ > 8.0 dS m^−1^ measured in a saturated paste extract (*c*. 80 mM NaCl) comprise < 0.5% of angiosperm species (1490/352 000 species), but are present in at least 33 orders and 110–120 families of flowering plants (The Plant List, 2013; Flowers *et al*., 2016). It has been suggested that halophytism is an evolutionarily labile character that has arisen independently in many angiosperm lineages from pre‐adapted genotypes (Flowers *et al*., 2010; Kadereit *et al*., 2012; Saslis‐Lagoudakis *et al*., 2014; Bromham, 2015; Cheeseman, 2015). Families with a large proportion of halophytes (> 10% of species in a family) occur in the Alismatales, Brassicales, Caryophyllales, Ericales, Fabales, Malphigiales, Piperales, Poales, Sapindales and Saxifragales (Saslis‐Lagoudakis *et al*., 2014; Flowers *et al*., 2016).

Halophytes can also be grouped into ‘ionotypes’, which are defined as characteristic ionomic features of plant species that are conserved in diverse environments (Albert & Popp, 1977; Gorham *et al*., 1980; Albert *et al*., 2000; Flowers & Colmer, 2008; White *et al*., 2012). Commelinid monocots (e.g. Poaceae, Cyperaceae, Juncaceae) are classed as ‘Na‐excluders’ and generally exhibit lower shoot Na concentrations ([Na]_shoot_) than other angiosperms growing in the same environment and Na/K quotients less than unity, whereas many eudicots are characterized by comparatively large [Na]_shoot_ and tissue Na/K quotients greater than unity (Albert & Popp, 1977; Gorham *et al*., 1980; Albert, 1982; Flowers & Colmer, 2008; Yang *et al*., 2012). Several families in the Caryophyllales (Amaranthaceae (Chenopodioideae), Caryophyllaceae, Tamaricaceae) exhibit exceptionally large [Na]_shoot_ and tissue Na/K quotients when grown in saline environments (Albert & Popp, 1977; Gorham *et al*., 1980; Albert, 1982; Flowers & Colmer, 2008; Yang *et al*., 2012; Zhang *et al*., 2012). It has also been observed that some Caryophyllales species have exceptionally large [Na]_shoot_, even when grown in nonsaline environments (Collander, 1941; Patel *et al*., 1980; Glenn & O'Leary, 1984; Broadley *et al*., 2004). For example, in a phylogenetically balanced study of the ionomes of 117 angiosperm species belonging to 25 orders grown hydroponically in a nonsaline solution containing 0.1 mM Na, it was noted that [Na]_shoot_ varied significantly among eudicot orders (*P *<* *0.05) and that three of the seven Caryophyllales species studied had conspicuously large [Na]_shoot_ (Broadley *et al*., 2004). It has been suggested that Na might have a special role in the biology of euhalophyte Caryophyllales, whose maximal growth requires Na accumulation (Flowers & Colmer, 2008), and that the characteristic ionome of the Caryophyllales might reflect their unusual ecology (White *et al*., 2015). Although Caryophyllales species can inhabit a variety of biomes worldwide, they comprise a significant proportion of the flora of many deserts (Fahn & Cutler, 1992), coastal regions (Kadereit *et al*., 2012) and soils with unbalanced mineral composition for plant nutrition, such as gypseous (Moore *et al*., 2014) and ultramafic/serpentine (White & Pongrac, 2016) soils.

The Caryophyllales order comprises over 11 000 species currently partitioned into *c*. 700 genera and 38 families (The Plant List, 2013; APGIV, 2016). About 5% of species in the Caryophyllales are halophytes and the order contains 35–40% of all known halophytic angiosperm species (Flowers *et al*., 2010, 2016; Saslis‐Lagoudakis *et al*., 2014). Of the most populous families in the Caryophyllales (> 50 species), the halophytic character is particularly prevalent in the Amaranthaceae (17.3% species), Frankeniaceae (16.7% species) and Tamaricaceae (31.1% species). In contrast with observations on other angiosperm orders, the halophytic character appears to be rarely lost in Caryophyllales lineages, such as the Chenopodioideae and Tamaricaceae, once it has evolved (Bromham, 2015). It has been suggested that the halophytic character might evolve from ancestors with a general complement of stress‐tolerance traits that enable lineages to adapt to a wide range of environmental challenges (Kadereit *et al*., 2012; Saslis‐Lagoudakis *et al*., 2014; Bromham, 2015). It is therefore noteworthy that the Caryophyllales order contains many succulent species (Kadereit *et al*., 2012; Rozema & Schat, 2013), many species that possess salt glands, which are specialized multicellular structures that excrete salt onto the leaf surface, or bladder cells, which are modified trichomes that accumulate salt and then burst (Thomson *et al*., 1988; Fahn & Cutler, 1992; Salama *et al*., 1999; Flowers *et al*., 2010; LoPresti, 2014), many species exhibiting C_4_ and Crassulacean acid metabolism (CAM) photosynthetic pathways (Silvera *et al*., 2010; Sage *et al*., 2011; Kadereit *et al*., 2012), many species that hyperaccumulate potentially toxic elements (White & Pongrac, 2016) and many species adapted to arid (Ehleringer *et al*., 1997) or alkaline (Yang *et al*., 2012) environments. The C_4_ photosynthetic pathway has evolved many times within the Caryophyllales (Sage *et al*., 2011) and Kadereit *et al*. (2012) observed that the rate of gain of the C_4_ photosynthetic character was greater in salt‐tolerant Chenopodioideae lineages, which they attributed to shared adaptations between C_4_ photosynthesis and salt tolerance as part of a wider drought tolerance syndrome. A similar dependence of the evolution of C_4_ photosynthesis with succulence and coastal habitat was also observed (Kadereit *et al*., 2012). CAM has also evolved many times within the Caryophyllales and is associated with succulence and other traits enabling water use efficiency in arid or saline environments (Edwards & Ogburn, 2012).

The present study investigated the prevalence of ‘Na‐hyperaccumulator’ species, which exhibit abnormally large [Na]_shoot_ (> 4 mg Na g^−1^ dry matter (DM)) when grown in nonsaline conditions (< 20 mM Na^+^ in the rhizosphere solution), among the angiosperms in general and the Caryophyllales in particular. The prevalence of this phenomenon among angiosperms is currently unknown and this study provides an original insight to its occurrence and evolutionary origins within the Caryophyllales order. It was observed that only the Caryophyllales species *Atriplex hortensis* and *Beta vulgaris* of the 12 halophytic species studied, representing 10 angiosperm orders, behaved as Na‐hyperaccumulators when grown in compost. Similarly, when 334 angiosperm species representing 35 angiosperm orders were grown hydroponically in a nonsaline solution containing 0.1 mM Na, a disproportionate number of Caryophyllales species exhibited abnormally large [Na]_shoot_. The bimodal distribution of the log‐normal [Na]_shoot_ of species within the Caryophyllales suggested at least two distinct [Na]_shoot_ phenotypes within this order. Mapping the trait of Na‐hyperaccumulation in nonsaline environments onto the phylogenetic relationships between Caryophyllales families (Crawley & Hilu, 2012; Hernández‐Ledesma *et al*., 2015; Yang *et al*., 2015), and between subfamilies within the Amaranthaceae, suggested that the trait had evolved several times within this order: in an ancestor of the Aizoaceae, but not the Phytolaccaceae or Nyctaginaceae, in ancestors of several lineages formerly classified as Chenopodiaceae, but not in the Amaranthaceae *sensu stricto*, and possibly in ancestors of species within the Cactaceae, Portulacaceae, Plumbaginaceae, Tamaricaceae and Polygonaceae. It is possible that the ability to hyperaccumulate Na^+^ might benefit plants by providing an alternative osmoticum to K^+^, especially in environments with low K availability (White, 2013). Thus, Na‐hyperaccumulation might have served Caryophyllales during their evolution in overcoming the selection pressures associated with the colonization of arid or saline environments, which require succulence and water conservation (Fahn & Cutler, 1992; Nobel, 2003; Flowers & Colmer, 2008; Kadereit *et al*., 2012).

## Materials and Methods

### Responses of halophytic species from different angiosperm orders to salinity

Responses to salinity were studied in 12 halophytic species, from 10 angiosperm orders, catalogued in the eHALOPH Halophytes Database (Flowers *et al*., 2016). These comprised: *Ammi visnaga* (L.) Lam. (Apiaceae, Apiales), *Asparagus officinalis* L. (Asparagaceae, Asparagales), *Atriplex hortensis* L. (Amaranthaceae, Caryophyllales), *Beta vulgaris* L. (Amaranthaceae, Caryophyllales), *Casuarina cunninghamiana* Miq. (Casuarinaceae, Fagales), *Colubrina asiatica* (L.) Brongn. (Rhamnaceae, Rosales), *Hibiscus tilliaceus* L. (Malvaceae, Malvales), *Hordeum jubatum* L. (Poaceae, Poales), *Kosteletzkya virginica* (L.) C. Presl ex A. Gray (Malvaceae, Malvales), *Lobularia maritima* (L.) Desv. (Brassicaceae, Brassicales), *Plantago maritima* L. (Plantaginaceae, Lamiales) and *Scaevola crassifolia* Labill. (Goodeniaceae, Asterales). Species were chosen on the basis of their availability from suppliers and their ability to grow in the glasshouse. Seeds of all species were obtained from Chiltern Seeds (Wallingford, UK), except *C. cunninghamiana*,* H. tilliaceous*,* K. virginica* and *P. maritima*, which were obtained from Rareexoticseeds (Montreal, Canada), Kenni Koala's Aussie Seed Store (Australia), Floridawildflowers (Crescent City, FL, USA) and Scotia Seeds (Brechin, UK), respectively. Seeds were germinated in the dark at between 10°C and 25°C, according to species requirements, on the surface of filter paper moistened with deionized water. Once a radicle was observed, individual seedlings were transplanted to rockwool plugs (2.5 × 2.5 × 4 cm^3^; Grodan, Hedehusene, Denmark) held in plastic trays in a glasshouse compartment at The James Hutton Institute, Dundee (UK; latitude 56°27′26″N, longitude 3°4′17″W), in which the experiment was subsequently performed, and irrigated with tap water containing 0.14 mM Na. The glasshouse compartment maintained a maximum of 25°C by day and a minimum of 15°C at night using automatic venting and supplementary heating.

Established seedlings were transferred to pots containing 1 l Levington Professional compost (ICL, Ipswich, UK) before the experiment. Two sets of plants, with up to 12 replicate plants per species in each set, were exposed to either nonsaline or saline irrigation. Plants were irrigated with 100 ml solution wk^−1^. Plants receiving the nonsaline treatment were irrigated with tap water containing 0.14 mM Na. The experiment was initiated by increasing the NaCl concentration in the irrigation water of the saline treatment to 50 mM for the first week, then 150 mM for the second week and, finally, 300 mM for the third week. Plants were harvested on 12 December 2014, 3 wk after the first addition of NaCl to the saline irrigation water. The fresh weight (FW) of whole shoots was determined immediately, and then samples were dried in an oven at 70°C to a constant weight and their DM was determined. Dried samples were milled to a powder using a ball mill (C + N Laboratory Mill; Christy and Norris Ltd, Chelmsford, UK), digested using HNO_3_ in sealed tubes in a microwave oven (MARS Xpress, CEM Corporation, Matthews, NC, USA), cleared using hydrogen peroxide (H_2_O_2_) and analysed for Na concentration using inductively coupled plasma‐mass spectrometry (ICP‐MS; ELAN DRCe, PerkinElmer, Waltham, MA, USA), as described by White *et al*. (2012).

### Phylogenetic effects on shoot sodium concentrations in plants grown hydroponically in a nonsaline solution

Phylogenetic effects on shoot Na concentrations in angiosperm species were assessed by combining data from six glasshouse experiments in which plants were grown hydroponically using a Nutrient Film Technique (NFT), essentially as described by Broadley *et al*. (2003). The final dataset comprised 334 species from 35 orders (Supporting Information Table S1). In all experiments, seeds were germinated in the dark on the surface of filter paper moistened with deionized water at temperatures between 4°C and 25°C, depending on their requirements. Once a radicle was observed, individual seedlings were transplanted to rockwool plugs (2.5 × 2.5 × 4 cm^3^; Grodan) held in plastic trays and irrigated with tap water. Plastic trays were either placed in a weaning room at 25°C or in the glasshouse compartment in which the experiments were subsequently performed. Once seedlings were established, the rockwool plugs containing the plants were transferred to the NFT system. Whenever possible, two rockwool plugs constituted each replicate and up to six replicates were obtained for each plant species. For experiments at both Warwick‐HRI, Wellesbourne (UK; latitude 52°12′18″N, longitude 1°36′00″W) and The James Hutton Institute, the glasshouse maintained a maximum of 20°C by day and a minimum of 15°C at night using automatic venting and supplementary heating. The recirculating nutrient solution contained 2 mM Ca(NO_3_)_2_, 2 mM NH_4_NO_3_, 0.75 mM MgSO_4_, 0.5 mM KOH, 0.25 mM KH_2_PO_4_, 0.1 mM FeNaEDTA, 30 μM H_3_BO_3_, 25 μM CaCl_2_, 10 μM MnSO_4_, 3 μM CuSO_4_, 1 μM ZnSO_4_ and 0.5 μM Na_2_MoO_4_. This was adjusted daily to pH 6, with H_2_SO_4_, and solutions were replaced completely once or twice each week. Seedlings were harvested during the exponential growth phase, 18–73 d after transfer to the hydroponic system, depending on the plant growth rate. Whenever possible, shoots were separated into leaves and stems. The FW of whole shoots or leaves was determined immediately and then samples were dried in an oven at 70–80°C to a constant weight and their DM was determined. Dried samples were milled to a powder using a ball mill, acid digested and their Na concentrations were determined either by inductively coupled plasma‐emission spectrometry (JY24; Jobin‐Yvon, Longjumeau, France), as described by Broadley *et al*. (2003; Exps 1–4), or by ICP‐MS, as described by White *et al*. (2012; Exps 5, 6).

Exp 1, described by Broadley *et al*. (2004), was undertaken in a glasshouse compartment at Warwick‐HRI between July and October 2001 to survey calcium (Ca), potassium (K), magnesium (Mg), Na, organic‐N and phosphorus (P) concentrations in leaves of a phylogenetically balanced set of 117 angiosperm species belonging to 25 orders. Exps 2(A–C) were undertaken sequentially in a glasshouse compartment at Warwick‐HRI between May and November 2003 to survey Ca concentrations in leaves of Magnoliid and monocot orders, with replication at the taxonomic level of the family. Six species representing three Magnoliid orders, 54 species representing eight monocot orders and nine other angiosperm species were grown in this experiment. Exp 3, described by White *et al*. (2007), was undertaken in a glasshouse compartment at Warwick‐HRI between July and August 2004 to survey selenium (Se) concentrations in leaves of 35 angiosperm species chosen to represent the range of ecological strategies for Se accumulation reported in angiosperms. Exp 4, described by White *et al*. (2015), was undertaken in a glasshouse compartment at Warwick‐HRI between June and August 2004 to survey leaf concentrations of Ca and Mg in as many Caryophyllales families as possible, with replication at the taxonomic level of the genus. Forty‐six Caryophyllales species were studied, representing eight families and 29 genera, together with 33 other angiosperm species. Exp 5 was undertaken in a glasshouse compartment at The James Hutton Institute between July and October 2011 to survey leaf Ca and Mg concentrations in a range of serpentine and nonserpentine plant species. These included 28 Caryophyllales species and 35 other angiosperm species. Exp 6 was undertaken in a glasshouse compartment at The James Hutton Institute between July and November 2015 to survey leaf Ca and Mg concentrations in a range of Arecaceae species, with replication at the taxonomic level of the genus. Twenty‐three Arecaceae species were studied, representing six genera, together with 11 other angiosperm species. Each experiment had several species in common with other experiments, allowing cross comparisons (Table S1). In total, 53 species, representing 22 families and 15 orders, were grown in more than one experiment.

### Statistics

Data are expressed as the mean and SE or SD of *n* observations. Statistical differences between treatments were assessed for each species by Student's *t*‐test. Estimates of variation in [Na]_shoot_ were assigned between and within orders (*n *=* *35), families (*n *=* *79) and species (*n *=* *334) using analyses of variance (ANOVA). All statistical analyses were performed using R 3.3.0 (R Core Team, 2016) employing a linear model of: [Na]_shoot_ ~ Order + Family + Species.

## Results

Twelve halophytic angiosperm species were grown in compost in pots that were irrigated with either nonsaline or saline solution. The shoot FW of most of these species did not differ significantly between plants that were irrigated with nonsaline and saline solutions (Table 1). However, the shoot FWs of *A. officinalis* (*P *=* *0.0193) and *K. virginica* (*P *=* *0.0430) were lower in plants irrigated with saline solution than in those irrigated with nonsaline solution, whereas the shoot FWs of *A. hortensis* (*P *=* *0.0090) were greater in plants irrigated with saline solution than in those irrigated with nonsaline solution. Previous studies have also suggested that halophytic *Atriplex* species grow best under slightly saline conditions (Black, 1960; Wallace *et al*., 1973; Storey & Wyn Jones, 1979; Albert, 1982; Glenn & O'Leary, 1984; Redondo‐Gómez *et al*., 2007; Glenn *et al*., 2012; Norman *et al*., 2013).

**Table 1:** Shoot fresh weight (FW), dry matter (DM) and sodium concentration ([Na]_shoot_) of 12 halophytic angiosperm species grown in pots irrigated with either 100 ml nonsaline (0.14 mM Na) or saline (50–300 mM Na) solution wk^−1^

Treatment	Species	Family	Order	FW (g)	Dry matter (DM) (g)	[Na]_shoot_ (mg g^−1^ DM)
Nonsaline	*Hordeum jubatum* L.	Poaceae	Poales	4.66 ± 2.24 (*n *=* *3)	0.43 ± 0.33 (*n *=* *3)	0.21 ± 0.02 (*n *=* *3)
Nonsaline	*Asparagus officinalis* L.	Asparagaceae	Asparagales	6.36 ± 0.75 (*n *=* *10)	0.74 ± 0.18 (*n *=* *10)	0.38 ± 0.05 (*n *=* *10)
Nonsaline	*Hibiscus tilliaceus* L.	Malvaceae	Malvales	2.87 (*n *=* *1)	0.19 (*n *=* *1)	0.69 (*n *=* *1)
Nonsaline	*Colubrina asiatica* (L.) Brogn.	Rhamnaceae	Rosales	1.47 ± 0.17 (*n *=* *2)	0.056 ± 0.003 (*n *=* *2)	0.73 ± 0.15 (*n *=* *2)
Nonsaline	*Casuarina cunninghamiana* Miq.	Casuarinaceae	Fagales	0.90 ± 0.20 (*n *=* *2)	0.045 ± 0.010 (*n *=* *2)	1.03 ± 0.24 (*n *=* *2)
Nonsaline	*Kosteletzkya virginica* (L.) C. Presl ex A. Gray	Malvaceae	Malvales	22.69 ± 3.75 (*n *=* *9)	2.09 ± 0.63 (*n *=* *9)	1.62 ± 0.13 (*n *=* *9)
Nonsaline	*Ammi visnaga* (L.) Lam.	Apiaceae	Apiales	22.56 ± 1.52 (*n *=* *8)	2.13 ± 0.18 (*n *=* *8)	2.16 ± 0.11 (*n *=* *8)
Nonsaline	*Lobularia maritima* (L.) Desv.	Brassicaceae	Brassicales	20.45 ± 8.68 (*n *=* *4)	1.10 ± 0.58 (*n *=* *4)	3.40 ± 0.27 (*n *=* *4)
Nonsaline	*Scaevola crassifolia* Labill.	Goodeniaceae	Asterales	49.53 ± 3.92 (*n *=* *4)	4.09 ± 0.27 (*n *=* *4)	3.78 ± 0.46 (*n *=* *4)
Nonsaline	*Plantago maritima* L.	Plantaginaceae	Lamiales	3.32 ± 0.52 (*n *=* *12)	0.053 ± 0.001 (*n *=* *12)	4.11 ± 0.33 (*n *=* *12)
Nonsaline	*Beta vulgaris* L.	Amaranthaceae	Caryophyllales	38.95 ± 7.28 (*n *=* *6)	2.46 ± 0.63 (*n *=* *6)	10.72 ± 1.03 (*n *=* *6)
Nonsaline	*Atriplex hortensis* L.	Amaranthaceae	Caryophyllales	28.18 ± 1.52 (*n *=* *7)	4.18 ± 0.47 (*n *=* *7)	12.02 ± 0.49 (*n *=* *7)
Saline	*Hordeum jubatum* L.	Poaceae	Poales	2.52 ± 0.86 (*n *=* *2)	0.13 ± 0.07 (*n *=* *2)	2.18 ± 0.27 (*n *=* *2)
Saline	*Asparagus officinalis* L.	Asparagaceae	Asparagales	4.11 ± 0.66 (*n *=* *9)	0.36 ± 0.15 (*n *=* *9)	2.66 ± 0.97 (*n *=* *9)
Saline	*Hibiscus tilliaceus* L.	Malvaceae	Malvales	3.36 (*n *=* *1)	0.17 (*n *=* *1)	4.10 (*n *=* *1)
Saline	*Colubrina asiatica* (L.) Brogn.	Rhamnaceae	Rosales	1.19 ± 0.36 (*n *=* *2)	0.054 ± 0.003 (*n *=* *2)	16.73 ± 10.32 (*n *=* *2)
Saline	*Casuarina cunninghamiana* Miq.	Casuarinaceae	Fagales	0.53 (*n *=* *1)	0.060 (*n *=* *1)	3.62 (*n *=* *1)
Saline	*Kosteletzkya virginica* (L.) C. Presl ex A. Gray	Malvaceae	Malvales	15.02 ± 1.58 (*n *=* *9)	1.66 ± 0.26 (*n *=* *9)	13.60 ± 0.94 (*n *=* *9)
Saline	*Ammi visnaga* (L.) Lam.	Apiaceae	Apiales	20.38 ± 1.65 (*n *=* *8)	1.96 ± 0.22 (*n *=* *8)	17.83 ± 1.14 (*n *=* *8)
Saline	*Lobularia maritima* (L.) Desv.	Brassicaceae	Brassicales	9.54 ± 1.75 (*n *=* *4)	0.49 ± 0.18 (*n *=* *4)	27.94 ± 2.30 (*n *=* *4)
Saline	*Scaevola crassifolia* Labill.	Goodeniaceae	Asterales	41.70 ± 9.77 (*n *=* *4)	3.41 ± 0.97 (*n *=* *4)	19.41 ± 1.97 (*n *=* *4)
Saline	*Plantago maritima* L.	Plantaginaceae	Lamiales	2.47 ± 0.37 (*n *=* *12)	0.052 ± 0.002 (*n *=* *12)	27.49 ± 1.01 (*n *=* *12)
Saline	*Beta vulgaris* L.	Amaranthaceae	Caryophyllales	35.72 ± 8.90 (*n *=* *6)	2.27 ± 0.60 (*n *=* *6)	29.56 ± 3.06 (*n *=* *6)
Saline	*Atriplex hortensis* L.	Amaranthaceae	Caryophyllales	32.99 ± 0.57 (*n *=* *6)	5.04 ± 0.24 (*n *=* *6)	32.44 ± 2.14 (*n *=* *6)

The experiment was initiated by increasing the NaCl concentration in the irrigation water of the saline treatment to 50 mM for the first week, then 150 mM for the second week and, finally, 300 mM for the third week. Plants were harvested 3 wk after the first addition of NaCl to the saline irrigation water. Data are expressed as mean ± SE of *n* observations.

The response of shoot Na concentration ([Na]_shoot_) to irrigation with saline solution differed between the species studied, and they could be classified into ‘Na‐excluder’, ‘Na‐responder’ and ‘Na‐accumulator’ species (cf. Baker, 1981). Of the 12 angiosperm species studied, four species appeared to exclude Na from their shoot tissues and had small [Na]_shoot_ when irrigated with either nonsaline or saline solution (Table 1). These ‘Na‐excluder’ species were the two monocot species studied, *H. jubatum* (Poales) and *A. officinalis* (Asparagales), *H. tilliaceus* (Malvales) and *C. cunninghamiana* (Fagales). Six species had relatively small [Na]_shoot_ when irrigated with nonsaline solution but, when irrigated with saline solution, their [Na]_shoot_ increased to > 10 mg g^−1^ DM. These ‘Na‐responder’ species were *C. asiatica* (Rosales), *K. virginica* (Malvales), *A. visnaga* (Apiales), *L. maritima* (Brassicales), *S. crassifolia* (Asterales) and *P. maritima* (Lamiales). The two Caryophyllales species studied, *B. vulgaris* and *A. hortensis*, both had exceptionally large [Na]_shoot_ when irrigated with nonsaline and saline solutions. These species could be designated as ‘Na‐accumulator’ species.

The constitutively large [Na]_shoot_ of ‘Na‐accumulator’ species could best be distinguished when plants were irrigated with nonsaline solution (Table 1). The distribution of this trait among angiosperms was therefore assessed by growing species hydroponically in a solution containing little Na, as described by Broadley *et al*. (2003). Data were combined from six individual glasshouse experiments (Table S1). As little of the variation in [Na]_shoot_ (3.4%) could be attributed to environment (i.e. experiment), the [Na]_shoot_ value for each species was calculated as the arithmetic mean of all experiments in which the species was grown (Table S1). The proportions of the variation in [Na]_shoot_ accounted for at the levels of order, family and species were 13.8%, 54.3% and 28.5%, respectively. This suggests that different plant families show distinct [Na]_shoot_ concentrations. Families with the largest mean [Na]_shoot_ of their constituent species were the Aizoaceae (24.47 ± 5.07 mg g^−1^ DM, *n *=* *7 species), Cactaceae (17.60 mg g^−1^ DM, *n *=* *1 species), Melastomataceae (5.23 mg g^−1^ DM, *n *=* *1 species), Portulacaceae (5.20 ± 4.60 mg g^−1^ DM, *n *=* *2 species) and Ericaceae (4.51 ± 3.89 mg g^−1^ DM, *n *=* *2 species). Three of these families are in the Caryophyllales order.

**Figure 1:**
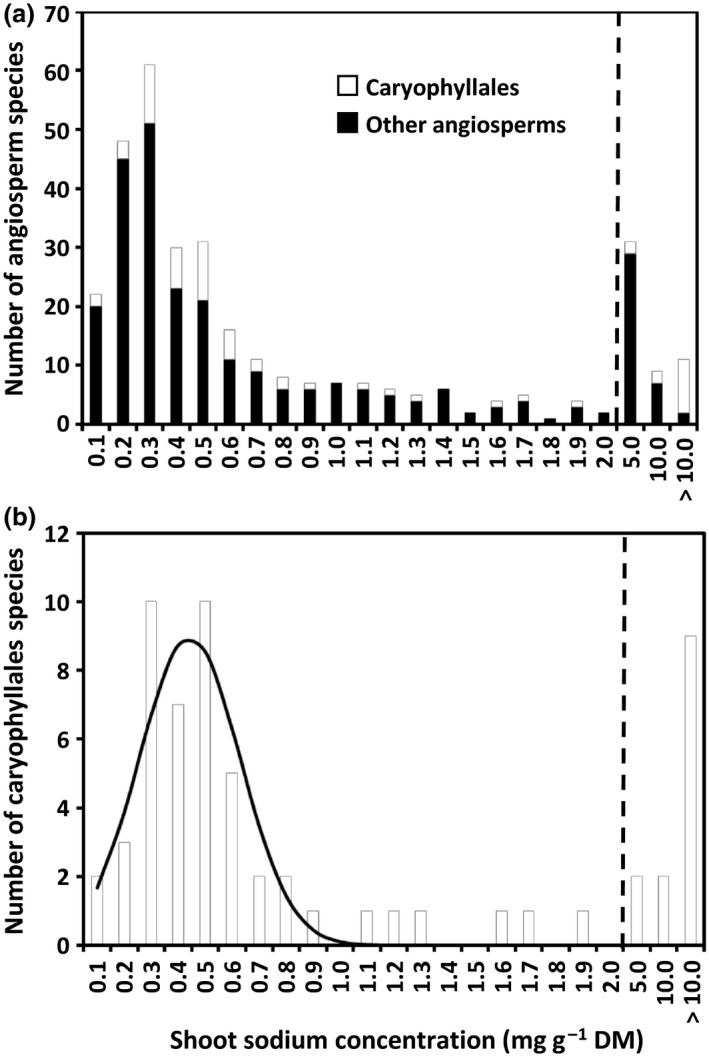
Frequency distributions of mean shoot sodium (Na) concentrations in (a) 334 species from 35 angiosperm orders and (b) 61 species from 10 Caryophyllales families, grown hydroponically in a nonsaline solution. The solid line indicates the normal (mean* *=* *0.393, SD **=* *0.185 mg Na g^−1^ DM, *n *=* *42 species) distribution fitted to the data from the 42 Caryophyllales species with the smallest shoot Na concentrations.

The [Na]_shoot_ value differed considerably between angiosperm species grown hydroponically in a nonsaline solution (Table S1; Fig. 1). Several species had mean [Na]_shoot_ greater than 10 mg g^−1^ DM. These species included nine Caryophyllales species, *B. vulgaris* (Amaranthaceae; 13.37 ± 2.35 mg g^−1^ DM, *n *=* *5 experiments), *Echinofossulocactus* sp. (Cactaceae; 17.60 mg g^−1^ DM, *n *=* *1 experiment), *Carpanthea pomeridiana* (Aizoaceae; 19.85 mg g^−1^ DM, *n *=* *1 experiment), *Hereroa odorata* (Aizoaceae; 20.17 mg g^−1^ DM, *n *=* *1 experiment), *Carpobrotus edulis* (Aizoaceae; 22.76 ± 3.31 mg g^−1^ DM, *n *=* *2 experiments), *A. hortensis* (Amaranthaceae; 23.75 ± 1.01 mg g^−1^ DM, *n *=* *2 experiments), *Stigmatocarpum criniflorum* (Aizoaceae; 30.81 ± 3.98 mg g^−1^ DM, *n *=* *3 experiments), *Mesembryanthemum cordifolium* (Aizoaceae; 37.42 ± 6.44 mg g^−1^ DM, *n *=* *2 experiments) and *Dorotheanthus bellidiformis* (Aizoaceae; 40.02 mg g^−1^ DM, *n *=* *1 experiment), and two other angiosperm species, C*allistemon rigidus* (Myrtaceae, Myrtales; 10.31 mg g^−1^ DM, *n *=* *1 experiment) and *Gladiolus carneus* (Iridaceae, Asparagales; 10.50 mg g^−1^ DM, *n *=* *1 experiment).

**Figure 2:**
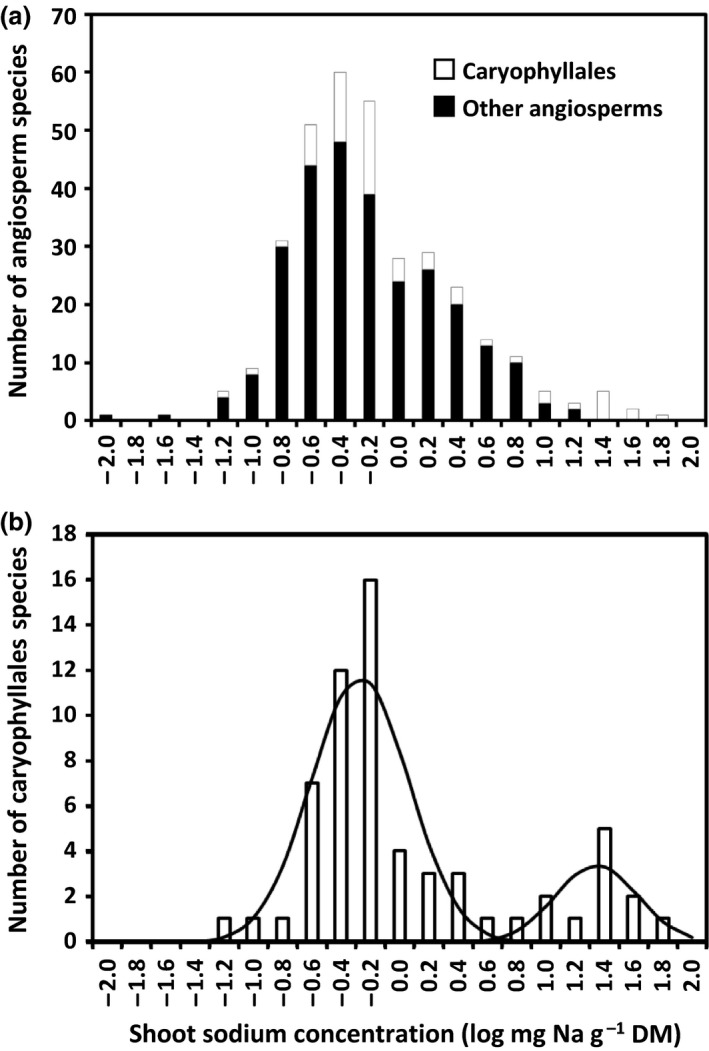
Frequency distributions of log‐normal mean shoot sodium (Na) concentrations in (a) 334 species from 35 angiosperm orders and (b) 61 species from 10 Caryophyllales families, grown hydroponically in a nonsaline solution. The solid line indicates two log‐normal distributions (first: mean* *=* *−0.3717, SD = 0.3299, *n *=* *49 species; second: mean = 1.246, SD = 0.2756, *n *=* *12 species) fitted to the data from the 49 Caryophyllales species with the smallest leaf Na concentrations and the 12 Caryophyllales species with the largest leaf Na concentrations, respectively.

The distribution of [Na]_shoot_ among the angiosperm species studied did not fit a simple normal distribution (Fig. 1a) and the log‐normal distribution of [Na]_shoot_ appeared to comprise the sum of at least three individual log‐normal distributions (Fig. 2a). The [Na]_shoot_ of Caryophyllales species differed by several orders of magnitude, from 0.05 mg g^−1^ DM in *Lewisia cotyledon* (Montiaceae) to 40.02 mg g^−1^ DM in *D. bellidiformis* (Aizoaceae). The distribution of [Na]_shoot_ among the Caryophyllales appeared to comprise a normal distribution (mean* *=* *0.393, SD* *= 0.185 mg g^−1^ DM, *n *=* *42 species) plus up to 19 species with abnormally large [Na]_shoot_ (Fig. 1b). The low probabilities of these species being part of the normal distribution suggested that there were at least two distinct [Na]_shoot_ phenotypes among Caryophyllales species. The species with [Na]_shoot_ at the limit for inclusion in the normal distribution were *Plumbago auriculata* (*P *=* *0.0153, rank no. 41), *Gomphrena serrata* (*P *=* *0.0106, rank no. 42), *Rumex hydrolapathum* (*P *=* *0.0003, rank no. 43) and *Limonium sinuatum* (*P *=* *0.0001, rank no. 44).

The distribution of log‐normal [Na]_shoot_ of Caryophyllales species appeared to comprise the sum of two discrete log‐normal distributions (Fig. 2b). The first log‐normal distribution (mean = −0.3717, SD* *=* *0.3299, *n *=* *49 species) contained 49 species and the second log‐normal distribution (mean* *=* *1.246, SD* *=* *0.2756, *n *=* *12 species) contained 12 species (Fig. 2b). As these two log‐normal distributions differed significantly (*P *<* *0.0001), these data suggest that there are at least two distinct [Na]_shoot_ phenotypes among Caryophyllales species. Considering the species with log [Na]_shoot_ at the extremes of these two distributions, the log [Na]_shoot_ of *Psylliostachys suworowi* had a greater probability of being in the first rather than the second log‐normal distribution (*P *=* *0.0076 vs *P *=* *0.0017), whereas the log [Na]_shoot_ of *Spergula arvensis* had a greater probability of being in the second rather than the first log‐normal distribution (*P *=* *0.0210 vs *P *=* *0.0007). The trait of abnormally large [Na]_shoot_ when plants are grown in nonsaline solutions will henceforth be termed ‘Na‐hyperaccumulation’, and the discrete set of 12 Caryophyllales species with large log [Na]_shoot_ are considered to be ‘Na‐hyperaccumulators’.

**Figure 3:**
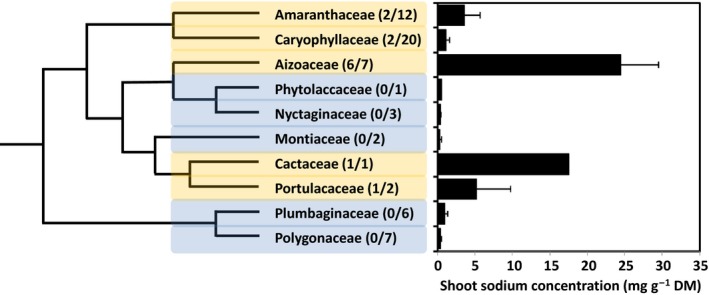
Phylogenetic relationships between 10 families of the Caryophyllales, based on the phylogeny derived by Crawley & Hilu (2012), and their shoot sodium concentrations ([Na]_shoot_). The number of species hyperaccumulating Na (numerator) and the number of species surveyed (denominator) are indicated in parentheses. Families with species expressing the trait of Na‐hyperaccumulation are highlighted in yellow and families without species expressing the trait of Na‐hyperaccumulation are highlighted in blue. Data are expressed as mean values with capped lines indicating the SE of the mean of the species surveyed.

The evolutionary origin of Na‐hyperaccumulation was sought by comparing the number of Na‐hyperaccumulator species and the mean [Na]_shoot_ in different families of the Caryophyllales (Fig. 3). The 12 Caryophyllales species exhibiting Na‐hyperaccumulation were distributed across five of the 10 Caryophyllales families represented in this study. However, the trait was most prevalent in the Aizoaceae. Six of the seven Aizoaceae species studied exhibited Na‐hyperaccumulation. These six species were among the seven Caryophyllales species with the largest [Na]_shoot_ (Table S1). Consequently, the Aizoaceae had the largest mean [Na]_shoot_ (24.47 ± 5.07 mg g^−1^ DM, *n *=* *7 species) of all the Caryophyllales families. The only Cactaceae species studied, *Echinofossulocactus* sp., also had one of the largest [Na]_shoot_ measured (17.60 mg g^−1^ DM, *n *=* *1 experiment). In addition, two of the 12 Amaranthaceae species studied (*A. hortensis*,* B. vulgaris*), two of the 20 Caryophyllaceae species studied (*Silene armeria*,* S. arvensis*) and one of the two Portulacaceae species studied (*Portulaca grandiflora*) could also be considered as Na‐hyperaccumulators (Table S1). However, as there were proportionally fewer Na‐hyperaccumulator species in these families, and the Na‐hyperaccumulator species in these families generally had smaller [Na]_shoot_ than the Aizoaceae Na‐hyperaccumulator species, their mean [Na]_shoot_ was less than the mean [Na]_shoot_ of the Aizoaceae (Fig. 3). No Na‐hyperaccumulator species were observed in the Phytolaccaceae, Nyctaginaceae, Montiaceae, Polygonaceae or Plumbaginaceae. Based on the phylogenetic relationships between Caryophyllales families proposed recently (Crawley & Hilu, 2012; Hernández‐Ledesma *et al*., 2015; Yang *et al*., 2015) and the data from the experiments reported here (Table S1), it appears that the trait of Na‐hyperaccumulation might have evolved several times within the Caryophyllales (Fig. 3). It is likely that the trait evolved in an ancestor of the Aizoaceae, but not the Phytolaccaceae or Nyctaginaceae. It is possible that the trait also evolved in ancestors of the Cactaceae and Portulacaceae, which are closely related (APGIV, 2016), and in ancestors of the Amaranthaceae and Caryophyllaceae.

## Discussion

Angiosperm species can be classified into ‘Na‐excluders’, ‘Na‐responders’ and ‘Na‐accumulators’ based on the relationship between their [Na]_shoot_ value and the salinity of the irrigation solution (cf Baker, 1981). This terminology, originally proposed to categorize the responses of plant species to toxic elements (‘heavy metals’) in the environment, also appears to be valid for Na accumulation, as the accumulation of excessive Na^+^ can be toxic to plants and plant species respond to Na^+^ in their environment either by excluding this cation or accumulating it safely in their tissues. Only two of the 12 halophytic species studied in detail in this article could be classified as Na‐accumulators (Table 1). These were the Caryophyllales species *A. hortensis* and *B. vulgaris*, which both had exceptionally large [Na]_shoot_ when irrigated with either nonsaline or saline solution. A similar response of [Na]_shoot_ to increasing salinity in the root environment has been observed previously for other Caryophyllales species, including members of the *Atriplex*,* Salicornia* and *Suaeda* genera (Albert, 1982; Glenn & O'Leary, 1984). However, not all Caryophyllales species exhibit this trait, and the response of [Na]_shoot_ to increasing salinity in the root environment of, for example, the miohalophytes *Rumex dentatus* and *Limonium perezii* is typical of Na‐excluders, whereas the response of [Na]_shoot_ to increasing salinity in the root environment of, for example, *Sarcobatus vermiculatus* is reminiscent of Na‐responders (Glenn & O'Leary, 1984).

The prevalence of Na‐accumulator species, which exhibit abnormally large [Na]_shoot_ when grown under nonsaline conditions, was assessed by combining data from six glasshouse experiments in which 334 angiosperm species representing 35 angiosperm orders had been grown hydroponically in a nonsaline solution containing 0.1 mM Na for 18–73 d (Table S1). It was observed that a relatively large number of Caryophyllales species exhibited abnormally large [Na]_shoot_ (> 10 mg g^−1^ DM) when grown in nonsaline solution (Table S1; Fig. 1). The distribution of the log‐normal [Na]_shoot_ of Caryophyllales species appeared to comprise two discrete log‐normal distributions containing 49 and 12 species, respectively (Fig. 2), suggesting that there are at least two distinct [Na]_shoot_ phenotypes among Caryophyllales species. The [Na]_shoot_ distinguishing between these two distributions was *c*. 4 mg Na g^−1^ DM.

The ability of plants to accumulate Na when growing in nonsaline environments is not considered to be an evolutionary advantage (Cheeseman, 2015). Indeed, it has been suggested that grazing by herbivores has selected for glycophyte species that maintain [Na]_shoot_ below *c*. 1–2 mg g^−1^ DM (Cheeseman, 2015). Nevertheless, it is possible that the ability to accommodate large [Na]_shoot_ might be an enabling trait allowing species to adapt to a variety of abiotic environmental challenges. It might confer the ability for osmotic adjustment in environments with low K phytoavailability or contribute to tolerance to arid or saline environments (Flowers & Colmer, 2008; Kadereit *et al*., 2012; White, 2013). However, it can be observed that the trait of Na‐hyperaccumulation within the Caryophyllales is not directly correlated with the expression of either C_4_ photosynthesis or CAM, tissue succulence, halophytism in general or the euhalophytic trait in particular (Table S2).

The evolutionary origins of the trait of abnormally large shoot Na accumulation when plants are grown in nonsaline solution, termed ‘Na‐hyperaccumulation’, can be investigated by mapping this trait on the phylogenetic relationships between Caryophyllales families (Crawley & Hilu, 2012; Hernández‐Ledesma *et al*., 2015; Yang *et al*., 2015). All Aizoaceae species appear to exhibit Na‐hyperaccumulation when grown in nonsaline environments (Table S2). Although *Delosperma cooperi* was not classified as an Na‐hyperaccumulator in the present study, it has previously been shown to accumulate > 4 mg Na g^−1^ DM shoot when grown in a peat substrate (Sunshine Mix #1, SunGro Hort., Bellevue, Washington, USA) and irrigated with tap water with an EC_e_ of 0.8 dS m^−1^ (Niu & Rodriguez, 2006). In addition to the species studied here, *Galenia pubescens* (Patel *et al*., 1980), *Galenia secunda* (Glenn & O'Leary, 1984), *Sesuvium portulacastrum* (Ramani *et al*., 2006; Slama *et al*., 2007; Rabhi *et al*., 2012; Wang *et al*., 2012), *Sesuvium verrucosum* (Glenn & O'Leary, 1984) and *Tetragonia tetragonioides* (Yousif *et al*., 2010) have all been reported to accumulate > 4 mg Na g^−1^ DM shoot when grown under nonsaline conditions (Table S2). In this context, it is noteworthy that many Aizoaceae species possess bladder cells (Thomson *et al*., 1988; Flowers *et al*., 2010).

The trait of Na‐hyperaccumulation in nonsaline environments is less ubiquitously exhibited by Amaranthaceae species (Table S2, and references therein). However, it is exhibited by many species formerly classified as Chenopodiaceae. It is exhibited by the Betoideae, *B. vulgaris* and *Hablitzia tamnoides*, by some Camphorosmoideae (e.g. *Bassia hyssopifolia* and *Maireana brevifolia*), by many Chenopoidioideae including most, but not all, *Atriplex* and *Chenopodium* species, by *Corispermum hyssopifolium* and *Corispermum pallasii* ssp. *membranaceum*, by all the Salicornioideae studied, including several *Salicornia* and *Tecticornia* species, by many Salsoloideae, and all *Suaeda* species (Table S2). By contrast, the trait is not exhibited by any Amaranthaceae *sensu stricto* (Amaranthoideae, Gomphrenoideae), with the exception of *Ptilotus polystachyus*. Although many Amaranthaceae species possess bladder cells or salt glands (Thomson *et al*., 1988; Fahn & Cutler, 1992; Flowers *et al*., 2010; LoPresti, 2014), there does not appear to be a direct correlation between the presence of salt glands and the ability to hyperaccumulate Na in nonsaline environments (Table S2).

Few Caryophyllaceae species had large [Na]_shoot_ when grown hydroponically in nonsaline solutions, with only two of the 20 species examined in the present study (*S. armeria*,* S. arvensis*) exhibiting Na‐hyperaccumulation (Table S1; Fig. 1). This is consistent with previous studies (Sonneveld & Voogt, 1983; Kwon *et al*., 2005; Heo *et al*., 2007; Jeong *et al*., 2014). Several species in the Sarcobataceae (*S. vermiculatus*), Portulacaceae (*P. grandiflora*;* Portulaca oleracea*) and Cactaceae (*Carnegiea gigantea*,* Echinocactus grusonii*,* Echinofossulocactus* sp., *Opuntia ficus‐indica*) exhibit large [Na]_shoot_ when grown in nonsaline environments (Table S2, and references therein). However, it is clear from the literature that not all Cactaceae exhibit large [Na]_shoot_ when grown in nonsaline environments (Table S2; Nobel, 2003; Goodman *et al*., 2012). No species in the Phytolaccaceae, Nyctaginaceae, Montiaceae, Basellaceae or Simmondsiaceae exhibited the trait (Table S2, and references therein).

In the experiments reported here, no Na‐hyperaccumulator species were observed in the Plumbaginaceae or Polygonaceae (Table S1; Fig. 3). Nevertheless, several species in these families have been reported to accumulate large [Na]_shoot_ when grown in nonsaline environments (Table S2, and references therein). In addition, all six species of Tamaricaceae studied to date appear to accumulate large [Na]_shoot_ when grown in nonsaline environments (Patel *et al*., 1980; Ding *et al*., 2010; Li *et al*., 2010; Gorai & Neffati, 2011; Sghaier *et al*., 2015; Sharif & Khan, 2016). It is, perhaps, noteworthy that many species in the Plumbaginaceae and Tamaricaceae possess salt glands, whereas members of the Polygonaceae do not (Thomson *et al*., 1988; Fahn & Cutler, 1992; Salama *et al*., 1999; Flowers *et al*., 2010). Again, there does not appear to be a direct correlation between the occurrence of salt glands and the ability of a species to hyperaccumulate Na in nonsaline environments (Table S2).

In conclusion, phylogenetic relationships between Caryophyllales families suggest that the trait of Na‐hyperaccumulation in nonsaline environments has evolved several times within this order (Fig. 3). The data presented here suggest that the trait evolved in an ancestor of the Aizoaceae, but not the Phytolaccaceae or Nyctaginaceae. It is also likely that the trait also evolved in an ancestor of species formerly classified as Chenopodiaceae (subfamilies Betoideae, Chenopodioideae, Camphorosmoideae, Salsoloideae, Salicornioideae, Suaedoideae), but not the Amaranthaceae *sensu stricto* (subfamilies Amaranthoideae, Gomphrenoideae). In addition, it is possible that the trait evolved in ancestors of the Sarcobataceae, Portulacaceae, Cactaceae, Tamaricaceae, Plumbaginaceae and Polygonaceae, but further studies are required to explore these hypotheses. Future studies should focus on the elucidation of the evolutionary origin of Na‐hyperaccumulation in nonsaline environments among species formerly classified as Chenopodiaceae, among species in the Cactaceae and Portulacaceae, which are currently under‐represented in published studies, and among species in the Plumbaginaceae, Tamaricaceae and Polygonaceae, to determine the extent of the trait in these families.

## Author contributions

P.J.W., M.R.B. and H.A.E‐S. designed the study. H.C.B., A.T., J.A.T. and G.W. conducted the experiments. P.J.W., M.R.B. and K.N. compiled and analysed the data. The manuscript was drafted by P.J.W. with contributions from all the other authors.

## Supporting information

Please note: Wiley Blackwell are not responsible for the content or functionality of any Supporting Information supplied by the authors. Any queries (other than missing material) should be directed to the *New Phytologist* Central Office.

**Table S1** Shoot sodium concentrations in 334 species from 35 angiosperm orders grown hydroponically in a nonsaline solution containing 0.1 mM Na^+^ in at least one of six glasshouse experiments
**Table S2** Occurrence of sodium (Na)‐hyperaccumulator species, having shoot Na concentrations > 4 mg g^−1^ dry matter when grown in nonsaline environments, within the Caryophyllales order, together with their halophytic and photosynthetic characteristics
