# Supplementary material for: Evolutionary origins of abnormally large shoot sodium accumulation in nonsaline environments within the Caryophyllales
Source: New Phytol. 2016 Dec 5;214(1):284–93. doi: 10.1111/nph.14370 (PMC5396351; doi:10.1111/nph.14370)
Supplement: Supplementary file 2 [file NPH-214-284-s002.pdf]

New Phytologist

New Phytologist Supporting Information Legends and References

Article title: Evolutionary origins of abnormally large shoot sodium accumulation in nonsaline environments within the Caryophyllales

Authors: Philip J. White, Helen C. Bowen, Martin R. Broadley, Hamed A. El-Serehy, Konrad Neugebauer, Anna Taylor, Jacqueline A. Thompson and Gladys Wright

Article acceptance date: 31 October 2016

The following Supporting Information is available for this article:

Table S1 Shoot sodium concentrations ([Na_{shoot}) in 334 species from 35 angiosperm orders grown hydroponically for 18-73 d in a nonsaline solution containing 0.1 mM Na^+ in at least one of six glasshouse experiments. The arithmetic mean from all experiments in which a species was grown is also indicated. Data within each experiment are expressed as mean ± SD of *n* replicates. Summary data are expressed as mean ± SE of *n* experiments. The names, synonyms and taxonomic authorities of plant species were consistent with The Plant List (http://www.theplantlist.org/) in August 2016. Plant families and orders were ascribed according to The Angiosperm Phylogeny Group IV (2016). Summary data for families and orders are expressed as mean ± SE of *n* species. (See separate Excel file)

Table S2 Occurrence of sodium (Na)-hyperaccumulator species within the Caryophyllales order. The trait of Na-hyperaccumulation was defined as a shoot Na concentration ([Na_{shoot}) greater than 4 mg g^{-1} DM in plants grown in nonsaline environments (electrical conductivity <2 dS m^{-1} in a saturated paste extract or NaCl concentration <20 mM in the rhizosphere solution). Information on whether the species is listed in the eHALOPH database (Flowers *et al.*, 2016), the class of halophyte to which it belongs, the presence of salt glands or epidermal bladder cells, its photosynthetic pathway and life form are reported where these are known to the authors.

References

No. 1 **Alam MA, Juraimi AS, Rafii MY, Hamid AA, Aslani F, Hakim MA. 2016.** Salinity-induced changes in the morphology and major mineral nutrient composition of purslane (*Portulaca oleracea* L.) accessions. *Biological Research* **49:** 24.

No. 2 **Alarcon JJ, Morales MA, Torrecillas A, Sanchez-Blanco MJ. 1999.** Growth, water relations and accumulation of organic and inorganic solutes in the halophyte *Limonium latifolium* cv. Avignon and its interspecific hybrid *Limonium caspia* x *Limonium latifolium* cv. Beltlaard during salt stress. *Journal of Plant Physiology* **154:** 795-801.

No. 3 **Ben Hamed K, Chibani F, Abdelly C, Magne C. 2014.** Growth, sodium uptake and antioxidant responses of coastal plants differing in their ecological status under increasing salinity. *Biologia* **69:** 193-201.

No. 4 **Bhargava A, Shukla S, Srivastava J, Singh N, Ohri D. 2008.** Genetic diversity for mineral accumulation in the foliage of *Chenopodium* spp. *Scientia Horticulturae* **118:** 338-346.

No. 5 **Black RF. 1960.** Effects of NaCl on the ion uptake and growth of *Atriplex vesicaria* Heward. *Australian Journal of Biological Science* **13:** 249-266.

No. 6 **Bohner A. 2001.** Physiologie und futterbaulicher Wert des Ampfers. *Alpenländisches Expertenforum* **7:** 39-44.

No. 7 **Cao J, Lv XY, Chen L, Xing JJ, Lan HY. 2015.** Effects of salinity on the growth, physiology and relevant gene expression of an annual halophyte grown from heteromorphic seeds. *AoB Plants* 7: plv112.

No. 8 **Chatterton NJ, McKell CM, Bingham FT, Clawson WJ. 1970.** Absorption of Na, Cl, and B by desert saltbush in relation to composition of nutrient solution culture. *Agronomy Journal* **62:** 351-352.

No. 9 **Chen L-H, Zhang B, Xu Z-Q. 2008.** Salt tolerance conferred by overexpression of *Arabidopsis* vacuolar Na^+/H^+ antiporter gene *AtNHX1* in common buckwheat (*Fagopyrum esculentum*). *Transgenic Research* **17:** 121-132.

No. 10 **Collander R. 1941.** Selective absorption of cations by higher plants. *Plant Physiology* **16:** 691-720.

No. 11 **Colmer TD, Vos H, Pedersen O. 2009.** Tolerance of combined submergence and salinity

in the halophytic stem-succulent *Tecticornia pergranulata*. *Annals of Botany* **103:** 303-312.

No. 12 **Daoud S, Harrouni C, Huchzermeyer B, Koyro H-W. 2008.** Comparison of salinity tolerance of two related subspecies of *Beta vulgaris*: the sea beet (*Beta vulgaris* ssp. *maritima*) and the sugar beet (*Beta vulgaris* ssp. *vulgaris*). In: Abdelly C, Öztürk M, Ashraf M, Grignon C, eds. *Biosaline agriculture and high salinity tolerance*. Basel, Switzerland: Birkhäuser, 115-129.

No. 13 **Dashti H, Azarnivand H, Shirani H, Hajabbasi MA, Maddahosseini SH. 2011.** Response of three *Calligonum* species to salinity at germination and seedling stages. *African Journal of Agricultural Research* **6:** 4487-4493.

No. 14 **Ding X, Tian C, Zhang S, Song J, Zhang F, Mi G, Feng G. 2010.** Effects of NO_3^--N on the growth and salinity tolerance of *Tamarix laxa* Willd. *Plant and Soil* **331:** 57-67.

No. 15 **Donovan LA, Richards JH, Muller MW. 1996.** Water relations and leaf chemistry of *Chrysothamnus nauseus* ssp. *consimilis* (Asteraceae) and *Sarcobatus vermiculatus* (Chenopodiaceae). *American Journal of Botany* **83:** 1637-1646.

No. 16 **English JP, Colmer TD. 2011.** Salinity and waterlogging tolerances in three stem-succulent halophytes (*Tecticornia* species) from the margins of ephemeral salt lakes. *Plant and Soil* **348:** 379-396.

No. 17 **Eshel A. 1985.** Effects of NaCl and KCl on growth and ionic composition of the halophytic C_4 succulent chenopods *Salsola kali*, *Suaeda monoica* and *Suaeda aegyptiaca*. *Australian Journal of Plant Physiology* **12:** 319-328.

No. 18 **Farrokhmanesh N, Golparvar AR, Naderi D. 2015.** Diversity of morphological and chemical traits and assessment of salinity tolerance in medicinal *Portulaca oleracea* L. genotypes. *Journal of Biodiversity and Environmental Sciences* **6:** 40-47.

No. 19 **Francois LE. 1986.** Salinity effects on four arid zone plants (*Parthenium argentatum*, *Simmondsia chinensis*, *Kochia prostrata* and *Kochia brevifolia*). *Journal of Arid Environments* **11:** 103-109.

No. 20 **Glenn EP, Brown JJ. 1998.** Effects of soil salt levels on the growth and water use efficiency of *Atriplex canescens* (Chenopodiaceae) varieties in drying soil. *American Journal of Botany* **85:** 10-16.

No. 21 **Glenn EP, O'Leary JW. 1984.** Relationship between salt accumulation and water content

of dicotyledonous halophytes. *Plant, Cell and Environment* **7:** 253-261.

No. 22 **Glenn EP, Nelson SG, Ambrose B, Martinez R, Soliz D, Pabendinskas V, Hultine K. 2012.** Comparison of salinity tolerance of three *Atriple*x spp. in well-watered and drying soils. *Environmental and Experimental Botany* **83:** 62-72.

No. 23 **Goodall DW. 1949.** Studies in the diagnosis of mineral deficiency. VI. The composition of weed leaves in relation to potassium deficiency in barley. *Annals of Applied Biology* **36:** 352-365.

No. 24 **Goodman J, Maschinski J, Hughes P, McAuliffe J, Roncal J, Powell D, Sternberg LO. 2012.** Differential response to soil salinity in endangered key tree cactus: implications for survival in a changing climate. *PLoS ONE* **7:** e32528.

No. 25 **Gorai M, Neffati M. 2011.** Osmotic adjustment, water relations and growth attributes of the xero-halophyte *Reaumuria vermiculata* L. (Tamaricaceae) in response to salt stress. *Acta Physiologiae Plantarum* **33:** 1425-1433.

No. 26 **Greenway H, Gunn A, Thomas DA. 1966.** Plant response to saline substrates. VIII Regulation of ion concentrations in salt-sensitive and halophytic species. *Australian Journal of Biological Sciences* **19:** 741-756.

No. 27 **Grieve CM, Poss JA, Grattan SR, Shouse PJ, Lieth JH, Zeng L. 2005.** Productivity and mineral nutrition of Limonium species irrigated with saline wastewaters. *HortScience* **40:** 654-658.

No. 28 **Gul B, Weber DJ, Khan MA. 2000.** Effect of salinity and planting density on physiological responses of *Allenrolfea occidentalis*. *Western North American Naturalist* **60:** 188-197.

No. 29 **Hasegawa E, Yoneyama T. 1995.** Effect of decrease in supply of potassium on the accumulation of cations in komatsuna and sugar beet plants. *Soil Science and Plant Nutrition* **41:** 393-398.

No. 30 **Hassan F, Ali E. 2014.** Effects of salt stress on growth, antioxidant enzyme activity and some other physiological parameters in jojoba [*Simmondsia chinensis* (Link) Schneider plant. *Australian Journal of Crop Science* **8:** 1615-1624.

No. 31 **Heidari-Sharifabada H, Mirzaie-Nodoushan H. 2006.** Salinity-induced growth and some metabolic changes in three *Salsola* species *Journal of Arid Environments* **67:** 715-720.

No. 32 **Heo EJ, Jung HH, Kim KS. 2007.** Response of *Dianthus japonicus* Thunb. to NaCl stress imposed at different growth stages. *Horticulture, Environment and Biotechnology* **48:** 381-386.

No. 33 **Jeong J-H, Kim S, Lee J-H,Choi W-Y, Lee K-B, Cho K-M. 2014.** Germination and growth response of *Spergularia marina* Griseb by salt concentration. *Korean Journal of Crop Science* **59:** 139-143.

No. 34 **Jia J, Cui X, Wu J, Wang J, Wang G. 2011.** Physiological and biochemical responses of halophyte *Kalidium foliatum* to salt stress. *African Journal of Biotechnology* **10:** 11468-11476.

No. 35 **Jin H, Dong D, Yang Q, Zhu D. 2016.** Salt-responsive transcriptome profiling of *Suaeda glauca* via RNA sequencing. *PLoS ONE* **11:** e0150504.

No. 36 **Kafi M, Rahimi Z. 2011.** Effect of salinity and silicon on root characteristics, growth, water status, proline content and ion accumulation of purslane (*Portulaca oleracea* L.). *Soil Science and Plant Nutrition* **57:** 341-347.

No. 37 **Khan MA, Ungar IA, Showalter AM. 2000a.** Effects of salinity on growth, water relations and ion accumulation of the subtropical perennial halophyte, *Atriplex griffithii* var. *stocksii*. *Annals of Botany* **85:** 225-232.

No. 38 **Khan MA, Ungar IA, Showalter AM. 2000b.** The effect of salinity on growth, water status and ion content of leaf succulent perennial halophyte, *Suaeda fruticosa* (L.) Forssk. *Journal of Arid Environments* 45: 73-84.

No. 39 **Khan MA, Ungar IA, Showalter AM. 2000c.** Effects of sodium chloride treatments on growth and ion accumulation of the halophyte *Haloxylon recurvum*. *Communications in Soil Science and Plant Analysis* **31:** 2763-2774.

No. 40 **Khan MA, Gul B, Weber DJ. 2001.** Effect of salinity on the growth and ion content of *Salicornia rubra*. *Communications in Soil Science and Plant Analysis* **32:** 2965-2977.

No. 41 **Khan MA, Ungar IA, Showalter AM. 2005.** Salt stimulation and tolerance in an intertidal stem-succulent halophyte. *Journal of Plant Nutrition* **28:** 1365-1374.

No. 42 **Kwon OK, Kim YA, Kim KS, Shin HK. 2005.** Growth and ion balance of carnation under salt stress. *Journal of the Korean Society for Horticultural Science* **46:** 38-384.

No. 43 **Langelaan JG, Troelstra SR. 1992.** Growth, chemical composition, and nitrate reductase activity of *Rumex* species in relation to form and level of N supply. *Plant and Soil* **145:** 215-229.

No. 44 **Li W, Khan A, Zhang X, Liu X. 2010.** Rooting and shoot growth of stem cuttings of saltcedar (*Tamarix chinensis* Lour) under salt stress. *Pakistan Journal of Botany* **42:** 4133-4142.

No. 45 **Liu A-R, Zhang Y-B, Chen Q-Y, Zhang C-Y, Xiong Z-L, He Q-Y, Wang G-Q. 2007.** Effects of salt stress on the growth and the photosynthesis in *Alternanthera philoxeroides* (Amaranthaceae). *Acta Botanica Yunnanica* **29:** 85-90.

No. 46 **Liu X, Duan D, Li W, Tadano T, Khan MA. 2008.** A comparative study on responses of growth and solute composition in halophytes *Suaeda salsa* and *Limonium bicolor* to salinity. In: Khan MA, Weber DJ, eds. *Ecophysiology of high salinity tolerant plants*, Dordrecht, Netherlands: Springer, 135-143.

No. 47 **Lv S, Jiang P, Chen X, Fan P, Wang X, Li Y. 2012.** Multiple compartmentalization of sodium conferred salt tolerance in *Salicornia europaea*. *Plant Physiology and Biochemistry* **51:** 47-52.

No. 48 **Matinzadeh Z, Breckle SW, Mirmassoumi M, Akhani H. 2013**. Ionic relationships in some halophytic Iranian Chenopodiaceae and their rhizospheres. *Plant and Soil* **372:** 523-539.

No. 49 **Meychik NR, Nikolaeva YI, Yermakov IP. 2013.** Physiological response of halophyte (*Suaeda altissima* (L.) Pall.) and glycophyte (*Spinacia oleracea* L.) to salinity. *American Journal of Plant Sciences* **4:** 427-435.

No. 50 **Moghaieb REA, Saneoka H, Fujita K. 2004.** Effect of salinity on osmotic adjustment, glycinebetaine accumulation and the betaine aldehyde dehydrogenase gene expression in two halophytic plants, *Salicornia europaea* and *Suaeda maritima*. *Plant Science* **166:** 1345-1349.

No. 51 **Morales MA, Olmos E, Torrecillas A, Sanchez-Blanco MJ, Alarcon JJ. 2001.** Differences in water relations, leaf ion accumulation and excretion rates between cultivated and wild species of *Limonium* sp. grown in conditions of saline stress. *Flora* **196:** 345-352.

No. 52 **Murillo-Amador B, CorteÂs-Avila A, Troyo-DieÂguez E, Nieto-Garibay A, Jones HG. 2001.** Effects of NaCl salinity on growth and production of young cladodes of *Opuntia ficus-indica*. *Journal of Agronomy and Crop Science* **187:** 269-279.

No. 53 **Nedjimi B, Daoud Y. 2009.** Effects of calcium chloride on growth, membrane permeability and root hydraulic conductivity in two *Atriplex* species grown at high (sodium chloride) salinity. *Journal of Plant Nutrition* **32:** 1818-1830.

No. 54 **Ning J, Ai S, Yang S, Cui L. Chen Y, Sun L, Wang R, Li M, Zeng Z. 2015.** Physiological and antioxidant responses of *Basella alba* to NaCl or Na_2SO_4 stress. *Acta Physiologiae Plantarum* **37:** 126.

No. 55 **Niu G, Rodriguez DS. 2006.** Relative salt tolerance of selected herbaceous perennials and groundcovers. *Scientia Horticulturae* **110:** 352-358.

No. 56 **Omueti O. 1982.** Effects of age on the elemental nutrients of *Celosia* cultivars. *Experimental Agriculture* **18:** 89-92.

No. 57 **Pan Y-Q, Guo H, Wang S-M, Zhao B, Zhang J-L, Ma Q, Yin H-J, Bao A-K. 2016.** The photosynthesis, Na^+/K^+ homeostasis and osmotic adjustment of *Atriplex canescens* in response to salinity. *Frontiers in Plant Science* **7:** 848.

No. 58 **Patel NT, Pandey AN. 2009.** Effect of soil salinity on growth, water status and nutrient accumulation in seedlings of *Suaeda nudiflora* (Chenopodiaceae). *Anales de Biología* **31:** 61-70.

No. 59 **Patel PM, Wallace A, Romney EM, Alexander GV. 1980.** A Collander-type experiment in large tanks of solution culture. *Journal of Plant Nutrition* **2:** 127-133.

No. 60 **Rabhi M, Castagna A, Remorini D, Scattino C, Smaoui A, Ranieri A, Abdelly C. 2012.** Photosynthetic responses to salinity in two obligate halophytes: *Sesuvium portulacastrum* and *Tecticornia indica*. *South African Journal of Botany* **79:** 39-47.

No. 61 **Ramani B, Reeck T, Debez A, Stelzer R, Huchzermeyer B, Schmidt A, Papenbrock J. 2006**. *Aster tripolium* L. and *Sesuvium portulacastrum* L.: two halophytes, two strategies to survive in saline habitats. *Plant Physiology and Biochemistry* **44:** 395-408.

No. 62 **Redondo-Gómez S, Mateos-Naranjo E, Davy AJ, Fernández-Muñoz F, Castellanos EM, Luque T, Figueroa ME. 2007.** Growth and photosynthetic responses to salinity of the salt-marsh shrub *Atriplex portulacoides*. *Annals of Botany* **100:** 555-563.

No. 63 **Reimann C. 1992.** Sodium exclusion by *Chenopodium* species. *Journal of Experimental Botany* **43:** 503-510.

No. 64 **Reimann C, Breckle S-W. 1993.** Sodium relations in Chenopodiaceae: a comparative approach. *Plant, Cell and Environment* **16:** 323-328.

No. 65 **Reimann C, Breckle S-W. 1995.** Salt tolerance and ion relations of *Salsola kali* L.: differences between ssp. *tragus* (L.) Nyman and ssp. *ruthenica* (Iljin) Soó. *New Phytologist* **130:**

37-48.

No. 66 **Rosen CJ, Bierman PM, Olson D. 1994.** Swiss chard and alfalfa responses to soils amended with municipal solid waste incinerator ash: Growth and elemental composition. *Journal of Agricultural Food Chemistry* **42:** 1361-1368.

No. 67 **Schuch UK, Kelly JJ. 2008.** Salinity tolerance of cacti and succulents. *Turfgrass, Landscape and Urban IPM Research Summary, P-155*. Tucson, AZ, USA: University of Arizona.

No. 68 **Sghaier DB, Duarte B, Bankaji I, Caçador I, Sleimi N. 2015.** Growth, chlorophyll fluorescence and mineral nutrition in the halophyte *Tamarix gallica* cultivated in combined stress conditions: Arsenic and NaCl. *Journal of Photochemistry and Photobiology B: Biology* **149:** 204-214.

No. 69 **Sharif F, Khan AU. 2016.** Effect of salinity on tissue nutrient contents of the four dryland tree species of Indus flood plains. *Arid Land Research and Management* **30:** 65-78.

No. 70 **Short DC, Colmer TD. 1999.** Salt tolerance in the halophyte *Halosarcia pergranulata* subsp. *pergranulata*. *Annals of Botany* **83:** 207-213.

No. 71 **Slama I, Ghnaya T, Savouré A, Abdelly C. 2008.** Combined effects of long-term salinity and soil drying on growth, water relations, nutrient status and proline accumulation of *Sesuvium portulacastrum*. *Comptes Rendus Biologies* **331:** 442-451.

No. 72 **Snowden RED, Wheeler BD. 1995.** Chemical changes in selected wetland plant species with increasing Fe supply, with specific reference to root precipitates and Fe tolerance. *New Phytologist* **131:** 503-520.

No. 73 **Sonneveld C, Voogt W. 1983.** Studies on the salt tolerance of some flower crops grown under glass. *Plant and Soil* **74:** 41-52.

No. 74 **Srivastava R. 2011.** Nutritional quality of some cultivated and wild species of *Amaranthus* L. *International Journal of Pharmaceutical Sciences and Research* **2:** 3152-3156.

No. 75 **Storey R, Wyn Jones RG. 1979.** Responses of *Atriplex spongiosa* and *Suaeda monoica* to salinity. *Plant Physiology* **63:** 156-162.

No. 76 **Suriyagoda LDB, Lambers H, Renton M, Ryan MH. 2012.** Growth, carboxylate exudates and nutrient dynamics in three herbaceous perennial plant species under low, moderate and high phosphorus supply. *Plant and Soil* **358:** 105-117.

No. 77 **Tauleigne-Gomes C, Lefèbvre C. 2008.** Natural hybridisation between two coastal endemic species of *Armeria* (Plumbaginaceae) from Portugal. 2. Ecological investigations on a hybrid zone. *Plant Systematics and Evolution* **273:** 225-236.

No. 78 **Taylor OO-A, Fetuga BL, Oyenuga VA. 1983.** Accumulation of mineral elements in five tropical leafy vegetables as influenced by nitrogen fertilization and age. *Scientia Horticulturae* **18:** 313-322.

No. 79 **Teixeira M, Carvalho IS. 2009.** Effects of salt stress on purslane (*Portulaca oleracea*) nutrition. *Annals of Applied Biology* **154:** 77-86.

No. 80 **Tremblin G, Ferard G. 1994.** Croissance et accumulation de sels chez *Halopeplis amplexicaulis* (Vahl.) Ung. cultivé à différentes salinités. *Acta Oecologica* **15:** 355-364.

No. 81 **Wallace A, Mueller RT, Romney EM. 1973.** Sodium relations in desert plants: 2. Distribution of cations in plant parts of three different species of *Atriplex*. *Soil Science* **115:** 390-394.

No. 82 **Wang D, Wang H, Han B, Wang B, Guo A, Liu C Chang L, Peng M, Wang X. 2012.** Sodium instead of potassium and chloride is an important macronutrient to improve leaf succulence and shoot development for halophyte *Sesuvium portulacastrum*. *Plant Physiology and Biochemistry* **51:** 53-62.

No. 83 **Wang J, Meng Y, Li B, Ma X, Lai Y, Si E, Yang K, Xu X, Shang X, Wang H, Wang D. 2015.** Physiological and proteomic analyses of salt stress response in the halophyte *Halogeton glomeratus*. *Plant, Cell and Environment* **38**: 655-669.

No. 84 **White PJ, Broadley MR, Thompson JA, McNicol JW, Crawley MJ, Poulton PR, Johnston AE. 2012.** Testing the distinctness of shoot ionomes of angiosperm families using the Rothamsted Park Grass Continuous Hay Experiment. *New Phytologist* **196:** 101-109.

No. 85 **Wilson C, Read JJ, Abo-Kassem E. 2002.** Effect of mixed-salt salinity on growth and ion relations of a quinoa and a wheat variety. *Journal of Plant Nutrition* **25:** 2689-2704.

No. 86 **Yamada M, Kuroda C, Fujiyama H. 2016.** Function of sodium and potassium in growth of sodium-loving Amaranthaceae species. *Soil Science and Plant Nutrition* **62:** 20-26.

No. 87 **Yousif BS, Nguyen NT, Fukuda Y, Hakata H, Okamoto Y, Masaoka Y, Saneoka H. 2010.** Effect of salinity on growth, mineral composition, photosynthesis and water relations of two

vegetable crops; New Zealand spinach (*Tetragonia tetragonioides*) and water spinach (*Ipomoea aquatica*). *International Journal of Agriculture and Biology* **12:** 211-216.

No. 88 **Yuan JF, Tian CY, Feng G. 2010.** Effects of sodium on nitrate uptake and osmotic adjustment of *Suaeda physophora*. *Journal of Arid Land* **2:** 190-196.

No. 89 **Zeng Y, Li L, Yang R, Yi X, Zhang B. 2015.** Contribution and distribution of inorganic ions and organic compounds to the osmotic adjustment in *Halostachys caspica* response to salt stress. *Scientific Reports* **5:** e13639.

No. 90 **Zhang H, Zhang G, Lü X, Zhou D, Han X. 2015.** Salt tolerance during seed germination and early seedling stages of 12 halophytes. *Plant and Soil* **388:** 229-241.

No. 91 **Zhang Y-M, Nie H-L. 2011.** Effects of biological soil crusts on seedling growth and element uptake in five desert plants in Junggar Basin, western China. *Chinese Journal of Plant Ecology* **35:** 380-388.

No. 92 **Zhao K-F, Fan H, Song J, Sun M-X, Wang B-Z, Zhang SQ, Ungar IA. 2005.** Two Na^+ and Cl^- hyperaccumulators of the Chenopodiaceae. *Journal of Integrative Plant Biology* **47:** 311-318.

No. 93 **Zheljazkov VD, Warman PR. 2004.** Source-separated municipal solid waste compost applications to swiss chard and basil. *Journal of Environmental Quality* **33:** 542-552.

No. 94 **Zia S, Egan TP, Khan MA. 2008.** Growth and selective ion transport of *Limonium stocksii* Plumbaginacea under saline conditions. *Pakistan Journal of Botany* **40:** 697-709.

No. 95 **Zurayk R, Tabbarah D, Banbukian L. 1993.** Preliminary studies on the salt tolerance and sodium relations of common ornamental plants. *Journal of Plant Nutrition* **16:** 1309-1316.

No. 96 **Zurayk RA, Khoury NF, Baalbaki RZ, Talho SN. 2001.** Salinity-heavy metal interactions in four salt-tolerant plant species. *Journal of Plant Nutrition* **24:** 1773-1786.
